# Environmental Enrichment Improved Learning and Memory, Increased Telencephalic Cell Proliferation, and Induced Differential Gene Expression in *Colossoma macropomum*

**DOI:** 10.3389/fphar.2020.00840

**Published:** 2020-06-12

**Authors:** Patrick Douglas Corrêa Pereira, Ediely Pereira Henrique, Danillo Monteiro Porfírio, Caio César de Sousa Crispim, Maitê Thaís Barros Campos, Renata Melo de Oliveira, Isabella Mesquita Sfair Silva, Luma Cristina Ferreira Guerreiro, Tiago Werley Pires da Silva, Anderson de Jesus Falcão da Silva, João Batista da Silva Rosa, Dmitre Leonardo Ferreira de Azevedo, Cecília Gabriella Coutinho Lima, Cintya Castro de Abreu, Carlos Santos Filho, Domingos Luiz Wanderley Picanço Diniz, Nara Gyzely de Morais Magalhães, Cristovam Guerreiro-Diniz, Cristovam Wanderley Picanço Diniz, Daniel Guerreiro Diniz

**Affiliations:** ^1^Laboratório de Biologia Molecular e Neuroecologia, Instituto Federal de Educação, Ciência e Tecnologia do Pará, Bragança, Brazil; ^2^Laboratório de Investigação em Neurodegeneração e Infecção, Instituto de Ciências Biológicas, Hospital Universitário João de Barros Barreto, Universidade Federal do Pará, Belém, Brazil; ^3^ICA - Instituto Ciências Agrárias, Universidade Federal Rural da Amazônia, Capitão Poço, Brazil; ^4^Laboratório de Fisiologia Ambiental Aplicada, Universidade Federal do Oeste do Pará, Oriximiná, Brazil; ^5^Laboratório de Microscopia Eletrônica, Instituto Evandro Chagas, Belém, Brazil

**Keywords:** environmental enrichment, spatial learning, and memory, object recognition, telencephalic and tectal cell count, neurotranscriptomics, *Colossoma macropomum*

## Abstract

Fish use spatial cognition based on allocentric cues to navigate, but little is known about how environmental enrichment (EE) affects learning and memory in correlation with hematological changes or gene expression in the fish brain. Here we investigated these questions in *Colossoma macropomum* (Teleostei). Fish were housed for 192 days in either EE or in an impoverished environment (IE) aquarium. EE contained toys, natural plants, and a 12-h/day water stream for voluntary exercise, whereas IE had no toys, plants, or water stream. A third plus maze aquarium was used for spatial and object recognition tests. Compared with IE, the EE fish showed greater learning rates, body length, and body weight. After behavioral tests, whole brain tissue was taken, stored in RNA-later, and then homogenized for DNA sequencing after conversion of isolated RNA. To compare read mapping and gene expression profiles across libraries for neurotranscriptome differential expression, we mapped back RNA-seq reads to the *C. macropomum de novo* assembled transcriptome. The results showed significant differential behavior, cell counts and gene expression in EE and IE individuals. As compared with IE, we found a greater number of cells in the telencephalon of individuals maintained in EE but no significant difference in the tectum opticum, suggesting differential plasticity in these areas. A total of 107,669 transcripts were found that ultimately yielded 64 differentially expressed transcripts between IE and EE brains. Another group of adult fish growing in aquaculture conditions were either subjected to exercise using running water flow or maintained sedentary. Flow cytometry analysis of peripheral blood showed a significantly higher density of lymphocytes, and platelets but no significant differences in erythrocytes and granulocytes. Thus, under the influence of contrasting environments, our findings showed differential changes at the behavioral, cellular, and molecular levels. We propose that the differential expression of selected transcripts, number of telencephalic cell counts, learning and memory performance, and selective hematological cell changes may be part of Teleostei adaptive physiological responses triggered by EE visuospatial and somatomotor stimulation. Our findings suggest abundant differential gene expression changes depending on environment and provide a basis for exploring gene regulation mechanisms under EE in *C. macropomum*.

## Introduction

Spatial memory and learning as a function of hippocampal formation in Teleostei is an ancient feature of the vertebrate forebrain that has been conserved during the divergent evolution of different vertebrate groups (Salas et al., 2006; Vargas et al., 2006). Despite their phylogenetic distance and difference in habitat from humans Teleostei have been used as a model for cognitive tasks because they show functional and anatomical homologies with respect to other vertebrates (Sovrano et al., 2020). Thus, it seems likely that fish can be useful for the analysis of mechanisms of complex vertebrate learning (Gerlai, 2017).

*Colossoma macropomum* is economically important in Brazilian aquaculture fisheries and a key organism in Amazonian river ecosystems. Despite multi-faceted economic interest in this species, current knowledge of its genetic resources remains limited. Here, we generated a reference *de novo* neurotranscriptome for *C. macropomum* of individuals raised in small-scale contrasting environments. We assessed spatial learning and memory and estimated the number of telencephalic and tectum opticum cells using an optical fractionator. Bioinformatic analyses were conducted to detect transcripts expressed under the influence of an impoverished compared to an enriched environment (IE vs. EE).

Neural cell proliferation is associated with the maintenance of complex neural mechanisms such as learning and memory making the search for basic mechanisms associated with these functions essential for understanding them (Abrous and Wojtowicz, 2008; Ge et al., 2008; Perera et al., 2008). Captive animals in general exhibit cognitive decline without adequate conditions for their maintenance. In fact, intensive productive systems in many cases may not involve consideration of the environmental adaptations necessary to meet an animal’s requirements for adequate brain and physical development (Martins et al., 2012). Environmental sensory stimuli, such as those resulting from social interaction, physical exercise, and stress levels, modulate neural cell proliferation (Gould et al., 1997; Kempermann et al., 1997; Van Praag et al., 1999; Eadie et al., 2005; Mirescu and Gould, 2006). In contrast to an IE that limits stimulation from social interaction, EEs provide diverse stimuli including visuo-spatial stimulation, physical exercise, and social interactions among individuals of the same and different species (Rosenzweig et al., 1978).

Many studies have previously investigated the influences of these two types of environment on neural mechanisms, especially in the cerebral cortex and limbic system (Kempermann et al., 1997; Pham et al., 2002; Branchi et al., 2006; Rossi et al., 2006; Mora et al., 2007; Schloesser et al., 2010; Von Krogh et al., 2010a). These analyses showed that the sensory, motor, and social stimuli of the EE act on the brain, inducing plastic responses in the local and projection circuitry. These responses are related to higher levels of neurotrophins and to glial and neural plasticity, increasing encephalic cellular proliferation in a variety of neurogenic niches (Pham et al., 2002; Branchi et al., 2006; Rossi et al., 2006; Zhu et al., 2006; Mora et al., 2007; Angelucci et al., 2009).

In teleosts, neurogenesis occurs during adulthood, and the rate of neuronal proliferation increases with age, body mass, and length (Birse et al., 1980; Zupanc and Horschke, 1995; Zupanc, 2006; Zupanc, 2008). However, little is known about the relevance of external influences on the neurogenesis of teleosts (Von Krogh et al., 2010a). Although many previous reports used transcriptomic analysis to a variety of experimental questions in Teleostei (Sullivan et al., 2015; Kumar and Denslow, 2017; Sudhagar et al., 2018), only a few reports applied neurotranscriptomics to address questions in behavior (Sneddon et al., 2005; Wong et al., 2014; Wong et al., 2015) and none searched for potential correlations between spatial memory and learning, differential gene expression, telencephalic cellular proliferation, and hematological changes.

The *Colossoma macropomum* is a key organism in Amazonian rivers ecosystems and important in Brazilian aquaculture fisheries. Despite multi-faceted economic interest in this species, our current knowledge of its genetic resources species remains very limited. Previous transcriptomic analysis of the *C. macropomum* has been used to anticipate differential genes expression to climate scenarios foreseen by the IPCC (Prado-Lima and Val, 2016). Total RNA was isolated from the white muscle specimens and 32,512 genes were identified and mapped using the *Danio rerio* genome as a reference. However, the effects of environmental influences on gene expression, neuroplastic response, peripheral blood response, in a system of cultivation in captivity are not clear. Thus, the objective of this work was to evaluate the potential influence of EE on spatial memory and learning, hematological cell changes, and encephalic and tectal cell proliferation in association with differential transcript expression using brain tissue transcriptomic analysis.

## Material and Methods

### Environmentally Enriched and Impoverished Aquariums

*Colossoma macropomum* individuals were maintained in aquariums with contrasting environments to investigate potential environmental influences on differential expression of gene transcripts in association with learning and memory performance and with cellular proliferation of telencephalic and tectal cells. All guidelines recommended by the National Institutes of Health (Guide for the Care and Use of Laboratory Animals) were followed, and the experimental protocol was submitted and approved prior to study initiation by the Ethics Committee on Experimental Animal Research from the Institute of Biological Sciences, Federal University of Pará, Brazil, CEUA-UFPA 3249260617.

**Figure 1:**
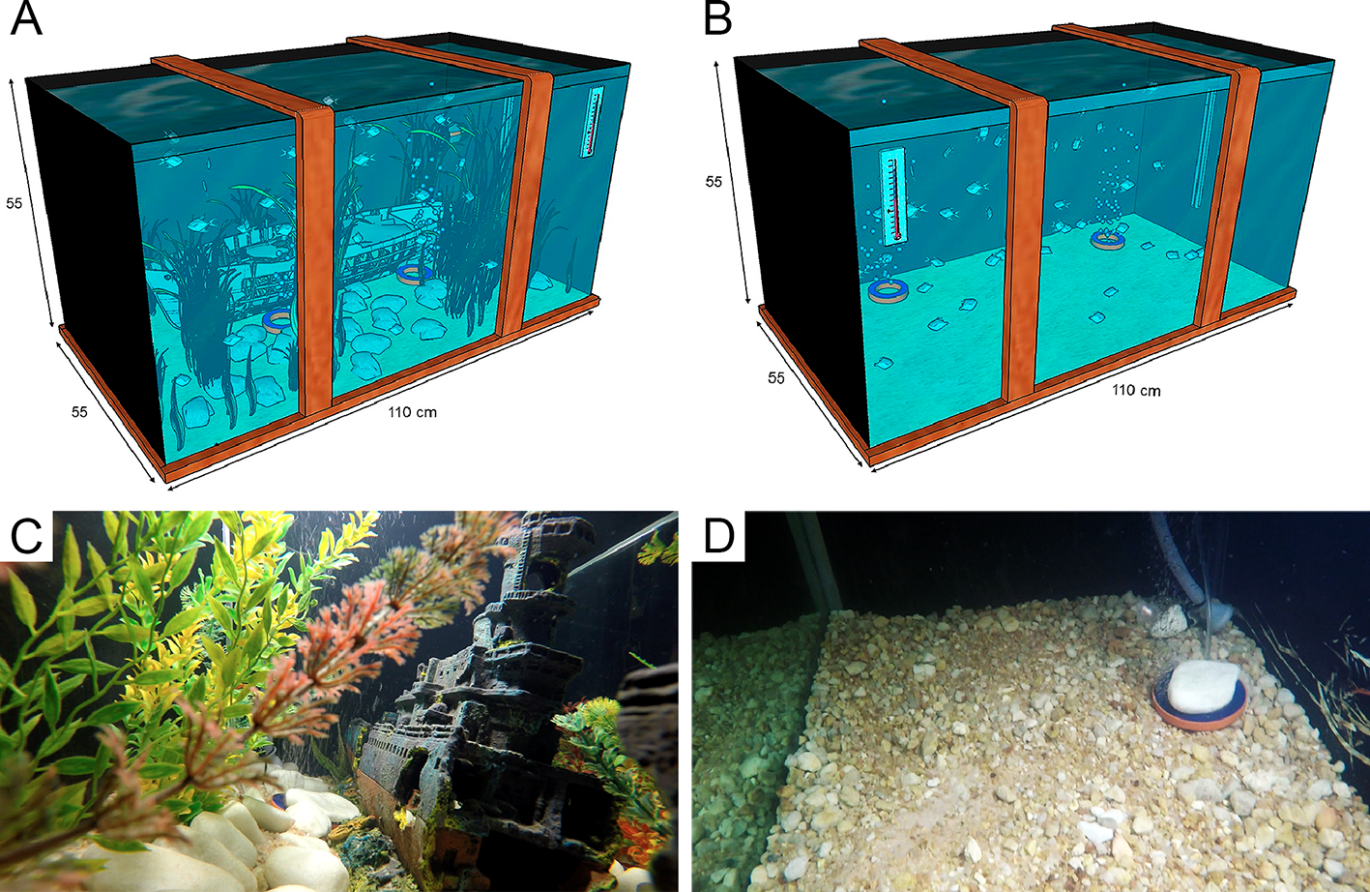
Small-scale enriched **(A**, **C)** and impoverished **(B**, **D)** aquaria. EE aquarium contains a running water flow pump for voluntary exercise and natural plants and a resin boat to provide rest and shelter. The IE aquarium did not contain any of these elements.

We reproduced small-scale EE with a water pump (Sunsun Wave Maker jvp-102b 5,000 l/h 110 V) that generated a running water flow for voluntary exercise and with natural plants and a resin boat to provide rest and shelter (**Figures 1A, C**). The IE aquarium did not contain any of these elements (**Figures 1B, D**).

We maintained each experimental group of juvenile fish (2.0–2.5 cm length) during 192 days in either the EE or IE aquarium (110 × 55 × 55 cm), each with a capacity of 332 L. Both aquaria contained biological filters (Sunsun Jp-025f 1,600 l/h), thermometers (Aquarium digital thermometer), stones, and an ultraviolet lamp and were set on a 12-h light/dark cycle. On alternate days, oxygen, pH, and temperature were measured and kept within acceptable standards for this species (oxygen ≥ 5.0 ppm, pH = 7.0, temperature 29°C ± 0.2°C). Mean values and standard errors for these features were as follows: oxygen (EE: 5.7 ± 0.04; AP: 5.3 ± 0.04 mg/L), pH (EE: 7.1 ± 0.05; IE: 6.99 ± 0.06), and temperature (EE: 28.8 ± 0.2; AP: 29.1± 0.15). *C. macropomum* shows normal growth at oxygen levels above 3 mg/L, which assure that the values adopted in this experiment (5.3–5.7 mg/L) perfectly supply the species requirement (Mendonça et al., 2012).

Twenty individuals (2.0–2.5 cm each at the beginning, and 9.0 to 10cm at the end of the experiment) in each aquarium were fed with the same amount (5 cm^3^) of granular food twice a day, with commercial feed (sera Pond Bio Granulat). No sexual identity was provided because all individuals at the age we did the experiment had their gonads undifferentiated (Lobo et al., 2020) and had not reached sexual maturity at the end. Concerning to sexual identity, our experiment was therefore blind, and the selected individuals were not from the same litter mate. No cortisol measurements were done and stocking density for EE = 1.25 and IE = 0.91 at the end of the experiment were below 1.97g/L as recommended by (Mendonça et al., 2012).

### Plus Maze Aquarium for Learning and Memory Testing

**Figure 2:**
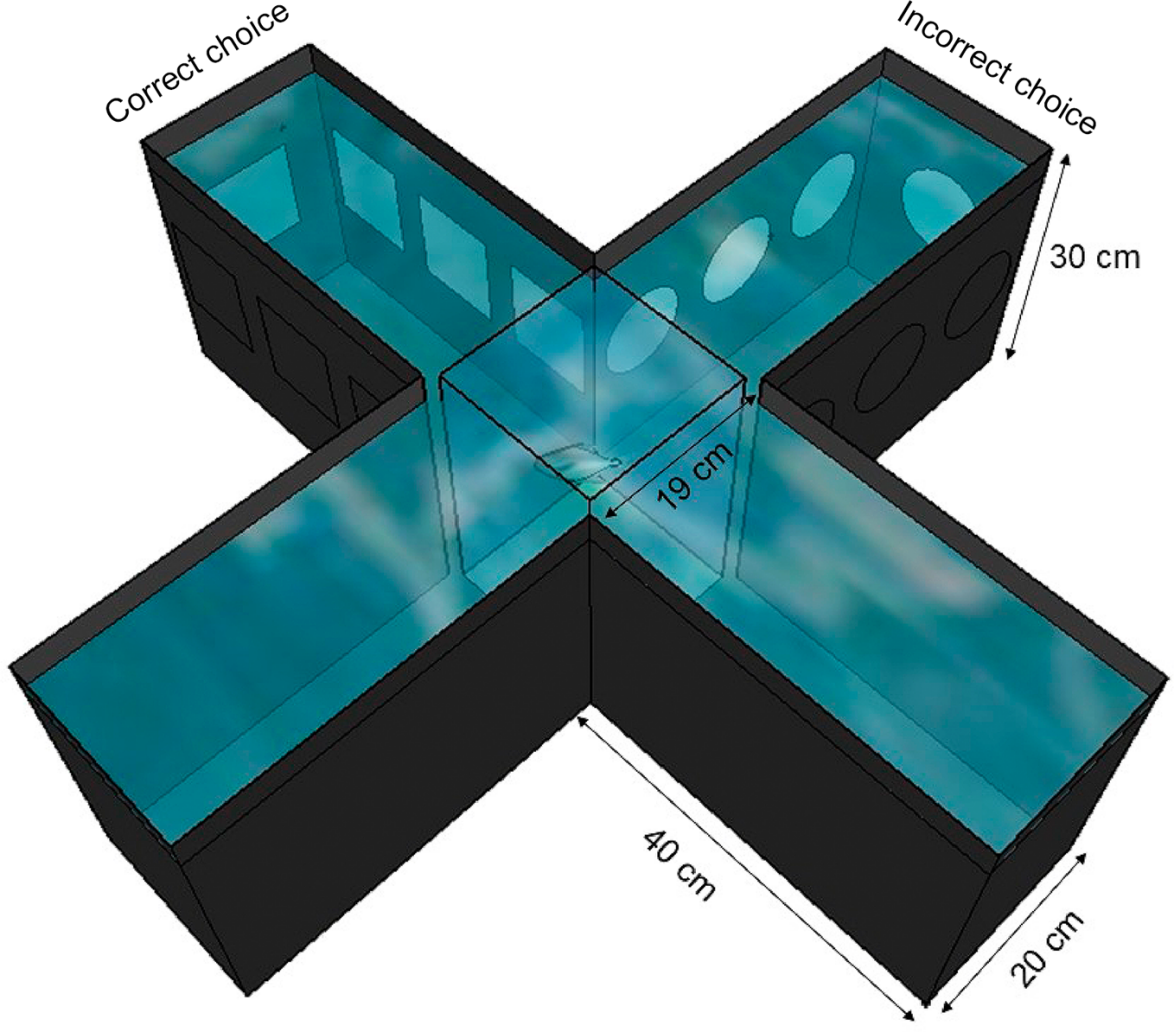
Plus maze test aquarium used to assess learning and memory performance. Visual cues are exhibited on two arms of the apparatus, where squares indicate the right choice, and circles the wrong one. Three consecutive correct choices or 7 correct choices in 10 attempts ended the test and the animal was removed from the cross aquarium and returned to its original aquarium.

In addition to EE and IE aquariums, we established a plus maze test apparatus (Sison and Gerlai, 2010) adapted to our experimental requirements (**Figure 2**) to assess spatial learning and memory performance.

The plus maze aquarium was maintained with similar water control parameters as described above. Visual cues were placed on two arms of the apparatus, with squares indicating the right choice and circles the wrong one. The test was performed over three consecutive days, during which each fish performed 10 trials in the apparatus, for a total of 30 sessions at the end of the third day if the right choice was not made earlier. All animals were adapted with an entrance into the apparatus during 5 min before the test began. The fish began the trials in the center of the apparatus, where they remained for 1 min inside a rectangular glass lid. The glass then was removed, and the animal had free access to the four arms of the apparatus. If the animal made a correct choice, it was removed immediately from the plus maze test aquarium and returned to its original aquarium. With a wrong choice, we imposed a 1-min restriction on the fish swimming space using a containment net at the bottom corners of the chosen arm. After application of this aversive following a wrong choice, these fish were transferred to their original aquarium. This restriction was systematically applied to all fish until they met the criteria for completing the task or the 30 sessions ended.

After the experiment, all fish from each treatment were euthanized using an overdose of Eugenol (clove oil, 0.40 mL/L) followed by craniectomy.

### Blood Samples for Flow Cytometry

Blood samples for flow cytometry were obtained directly from the heart by intracardiac puncture and stored in tubes containing EDTA anticoagulant. The blood samples were subjected to flow cytometry after rapid staining with fluorescent dyes at room temperature, based on previous methods published elsewhere (Valet, 1984). The fluorescent dyes mixture included 3,3-dihexyloxacarbocyanine, a dye sensitive to changes in the membrane potential (DiOC6, 19196KJV Sigma-Aldrich) and acridine orange (MKBS4724V, Sigma-Aldrich) for DNA/RNA staining as follows: 10 mg of DIOC6 was diluted in 1.74 ml of dimethyl sulfoxide; 400µg of Acridine orange (AO) was then added to 1 ml of this solution. Then, 40 µl of this cocktail of fluorescent dies was added to a mixture of 10 µl of blood in 1.95 µl of Dulbecco`s Phosphate Buffered Saline. Typical histograms and plotting of flow cytometric measurements of DiOC6/AO stained blood cells are obtained after subjecting the samples to cytometer and counts of the absolute and relative number of erythrocytes, reticulocytes, thrombocytes, lymphocytes, and granulocytes were obtained.

### Fixation and Histological Procedures

**Figure 3:**
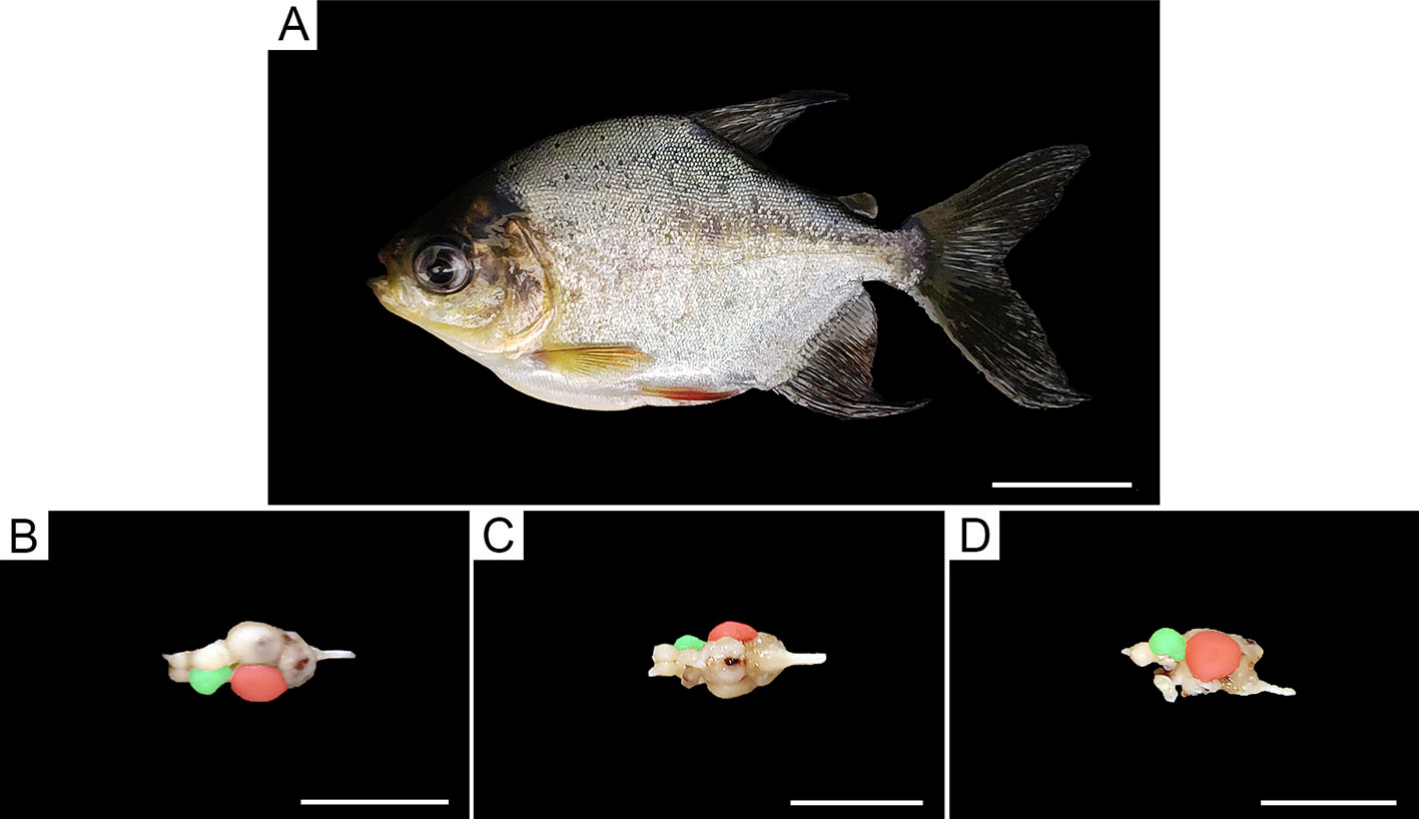
*C. macropomum* specimen **(A)** and brain in the dorsal **(B)**, ventral **(C)**, and lateral **(D)** views. Stereological cell counts were done in the telencephalon (green area) and tectum opticum (pink area). Scale bars: (A) = 2 cm, **(B**, **D)** = 1 cm.

After craniotomy, brains were fixed by immersion in buffered 10% formalin. **Figure 3** illustrates a *C. macropomum* specimen (**Figure 3A**) and corresponding dissected brain in the dorsal (**Figure 3B**), ventral (**Figure 3C**), and lateral (**Figure 3D**) views. The areas of interest for stereological cell counts were the telencephalon (green area) and tectum opticum (pink area). After 7 days, brains were dissected and cut using a vibratome. Serial sections 80-µm thick were collected, and 1:3 anatomical series were kept in the same fixative and maintained at 4°C in the fridge. Sections then were mounted on gelatinized glass slides, air dried, stained with cresyl violet, dehydrated, cleared, mounted with DPX Mountant, and coverslipped.

### Photomicrography

Digital photomicrographs were made with a digital camera (Microfire, Optronics, Fremont, CA, USA) attached to a Nikon microscope (Optiphot-2; Melville, NY, USA). The levels of brightness and contrast applied to the entire image were adjusted using Adobe Photoshop CC 2018 (San Jose, CA, USA).

### Stereology

**Figure 4:**
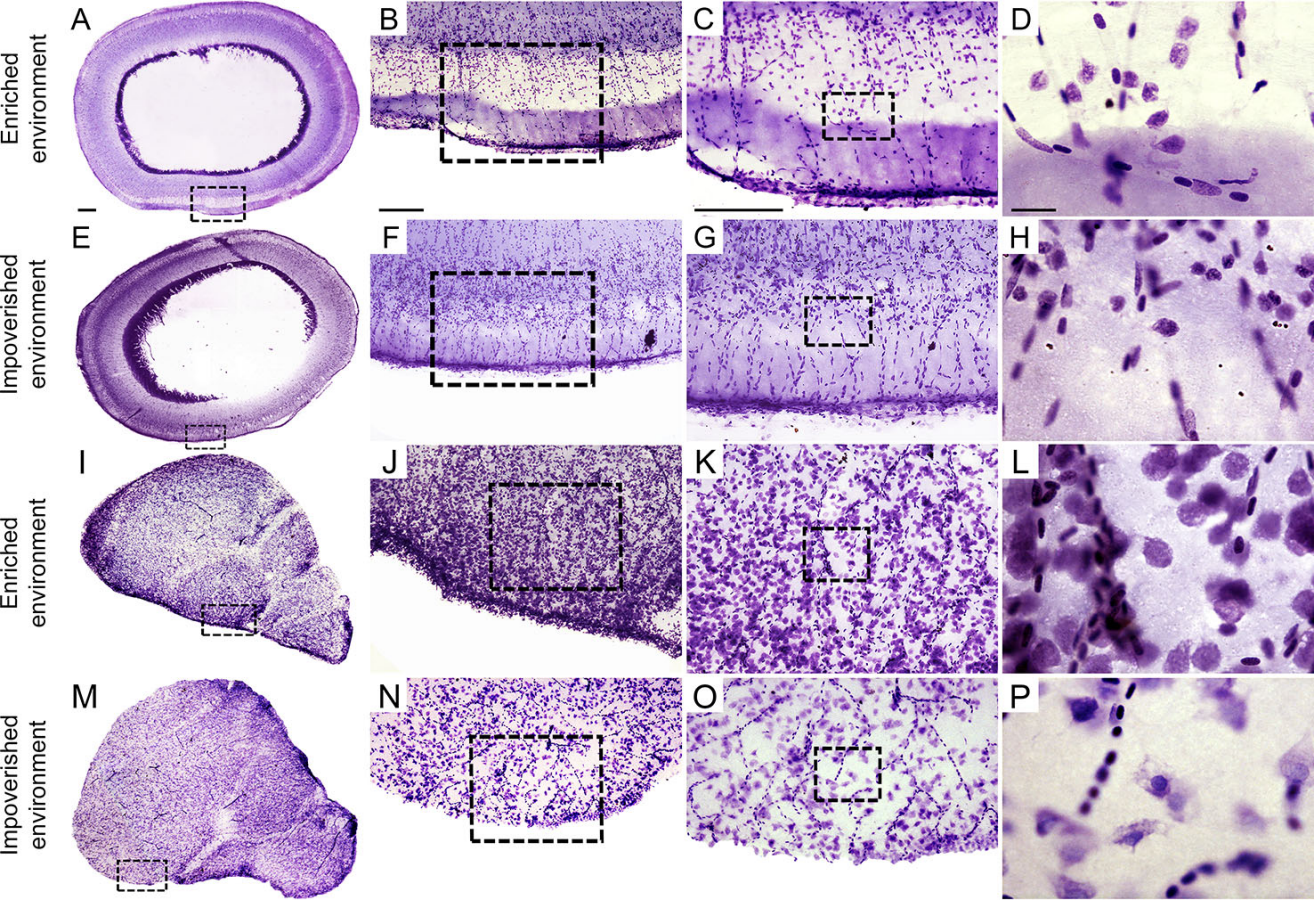
Tectal **(A**–**H)** and telencephalic **(I**–**P)** Nissl-stained parasagittal sections from *C. macropomum* to illustrate the area and objects of interest for stereological analysis. All scale bars, 250 µm, except **(D, H, L, P)** which are 25 µm.

To investigate the influences of each environment on total telencephalic cell numbers (neurons and glia), we compared the stereological estimates of the total cell counts from five individuals of each group, using the optical fractionator (West, 1999). All stereological analysis requires correct identification of the region of interest. As in all other teleosts investigated so far, the telencephalon of *C. macropomum* shows two solid hemispheres consisting of several nuclear masses and separated by a common ventricle (Ito and Yamamoto, 2009). The boundaries of the telencephalon are readily recognized in Nissl-stained sections of teleost fish (Ito and Yamamoto, 2009). To count cells, we used the systematic and random distribution of counting blocks in telencephalic parasagittal and optical tectum sections (**Figure 4**). **Figures 4A–P** are photomicrographs of the optical tectum and telencephalon under progressive magnifications to illustrate the area and the object of interest for stereological analysis. This sampling is a key step because it is not possible to count all cells within the region of interest. Avoiding this dilemma and obtaining estimates close to the real values requires use of systematic and random data collection. This alternative ensures adequate estimation of the total number of cells from the number of cells detected in each sampled counting box and in the sample probability (Schmitz and Hof, 2005). However, to minimize methodological errors, we also selected grid and counting box dimensions to generate, after counting procedures, a Scheaffer coefficient of error smaller than 0.05. Methodological errors ≤ 0.05 contribute little to the observed group variance. To fulfill this criterion, we conducted a pilot study to test different grid and counting box dimensions in telencephalic sections of *C. macropomum* and counted cells until we identified the appropriate coefficient of error, increasing precision of the estimate.

Photomicrographs taken at low, medium, and high power in **Figure 4** show tectal and telencephalic sections stained with cresyl violet. These images illustrate the microscopic views of areas and objects of interest for stereological analysis.

### Neurotranscriptomics Analysis: Extraction, Library, and RNA Sequencing

Fresh (no formalin-fixed) dissected brains, selected for transcriptomic analysis, were immersed in 10 volumes of tissue storage reagent (RNAlater^®^ Solution, Thermo Fisher Scientific) to permeate tissue, stabilize, and protect the integrity of RNA. All immersed tissue was stored for later analysis at −20°C. To broadly describe the brain-expressed transcriptome of C. macropomum, we extracted RNA from the whole brains of four individuals from each experimental group. Whole brain tissue stored in RNA-later was homogenized for DNA sequencing after conversion of isolated RNA. To compare read mapping and transcript expression profiles across samples, we mapped RNA-seq reads to the assembled *C. macropomum* transcriptome, which we used in the analysis of neurotranscriptome differential expression. For central nervous system (CNS) RNA extraction, we used the Dynabeads RNA DIRECT Micro Kit (Thermo Fisher Scientific) and followed the manufacturer’s suggested protocol for isolating RNA from tissues. For cDNA conversion, we used the Ion Total RNA-Seq Kit V2 kit (Life Technologies). Automated template preparation was done with the Ion Chef System (Thermo Fisher Scientific), following the manufacturer’s instructions. Sequencing of fragments was done with the Ion 540™ Chip, and we used the Ion S5™ System (Ion Torrent™) for reading the chip. Eight single end read FASTQ files were generated that reference the eight biological replicates (four individuals from the EE group and four from the IE group).

### Filtration and Trimming

To check the quality of sequences resulting from the sequencing process, we used FastQC software (Version 0.11.0; Bolger et al. (2014)). After quality checking, sequenced FASTQ files were filtered using Trimmomatic software, version 0.36, according to Macmanes (2014). **Table 1** shows the number of total trimmed reads per brain sample of juvenile *Colossoma macropomum*.

**Table 1:** Total number of trimmed reads per brain sample of juvenile *Colossoma macropomum*.

Tissue	Individual	Number of reads
Whole Brain	EE01	4,598,458
Whole Brain	EE10	2,281,003
Whole Brain	EE13	7,170,084
Whole Brain	EE19	2,000,769
Whole Brain	IE04	6,754,434
Whole Brain	IE07	4,210,463
Whole Brain	IE08	5,419,968
Whole Brain	IE10	7,614,932

### Re-Assembly of the Transcriptome Functional Annotation of Differentially Expressed Transcripts

To perform *de novo* transcriptome assembly, we used Trinity 2.8 software (Grabherr et al., 2011) following default parameters. To quantify the transcript expression, we used Salmon 0.14 (Patro et al., 2017) and to identify differentially expressed transcripts we followed the pipeline according Haas et al. (2013), we performed the transcript annotation protocol using Blast2GO v5.1 (Conesa et al., 2005), as follows: BLAST + (Altschul et al., 1990) to identify transcripts with the Uniprot SwissProt (UniProt Consortium, 2018) and non-redundant NCBI databases; InterproScan (Mitchell et al., 2019) to identify transcripts using available protein families; and association of ontology terms [gene ontology (GO) terms] with the BLAST hits. For visualization/creation of graphs related to genes and their respective ontology terms, we used the WEGO 2.0 tool (Ye et al., 2018).

### Statistical Analysis of Behavioral Data

To assess learning rate and memory, we used Kaplan-Meier survival curves, which predict time to an event in a series of declining horizontal steps showing learning rate as a function of time. A main obstacle to the widespread use of survival analysis (as in the present report) is the word “survival,” which can give the impression that its only use is for data related to death or failure (In and Lee, 2018). Here, we used it in the context of the following question: How long does it take for a fish to learn and remember the correct arm to choose (i.e., time to the occurrence of the correct choice versus the wrong choice)? The “event” was meeting the learning and memory criteria as a function of the progression of the training sessions (time). In our sample, every time a fish achieved three consecutive correct choices or seven correct choices in a total of 10 trials, the test was ended and the animal removed from the sample, made visible by a decrease in the learning rate (the curve stepdown). Thus, the Kaplan-Meier survival curve as used here illustrates the probability of the correct choice being made as a function of the number of training sessions. If individuals kept in one environment met the criteria for removal faster than those in the other environment, for example, their Kaplan-Meier curve would show a more rapid significant increase in learning rate. The log-rank test compares the curves and shows when two Kaplan-Meier curves are statistically equivalent, so we applied this test to evaluate the curves for significant differences between them.

### Biometric and Stereological Comparative Results

To compare the biometric and stereological results obtained in the two experimental groups, we used parametric and non-parametric statistical analysis to allow comparisons between normal and non-normal distributions. To test whether the distributions were normal or non-normal, we used the Shapiro-Wilk test.

## Results

### Body Length, Body Weight, and Behavior Analysis

**Figure 5:**
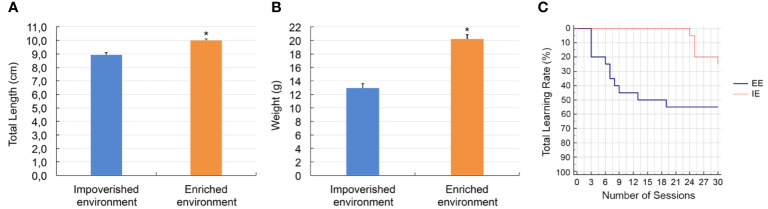
Length **(A)**, body weight **(B)**, and learning rates **(C)**, with significantly greater mean values for juvenile fish growing in the enriched aquaria. (*) indicates statistically significant differences. Kaplan-Meier curves **(C)** (log-rank test with a Chi-squared value of 58.6, degrees of freedom = 1, p = 0.01), showing statistically significant differences between the EE and IE learning rates.

**Figure 5** illustrates a comparative analysis of total body length (A), body weight (B), and learning rates (C) from fish maintained in EE (orange) and IE (blue). Compared with IE animals, the EE fish showed greater learning rates (Kaplan-Meier and log-rank test, p = 0.01), total body length (EE 9.99 ± 0.11 cm; IE 8.90 ± 0.14 cm; p < 0.0001; U = 230; Z (U) = 44.279), and weight (EE 20.20 ± 0.64 g; IE 14.88 ± 0.52 g; p < 0.0001; U = 169.5, Z (U) = 5.25). After 30 experimental training sessions, more than 50% of EE fish met the learning criteria. In contrast, less than 30% of those from the impoverished aquarium did so.

### Telencephalic and Tectal Stereological Analysis

**Figure 6:**
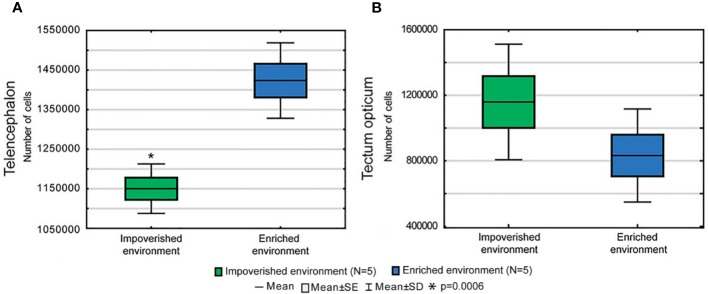
Total number of cells of the telencephalon **(A)** and tectum opticum **(B)** from individuals raised in enriched (EE) or in impoverished (IE) aquaria. The average total number of telencephalic cells was greater in EE than in IE animals. Non-significant differences were found in the tectum opticum.

Total cell counts differed significantly in the telencephalon but not in the tectal region of EE and IE animals suggesting that contrasting visuo-spatial and somatomotor stimulations of enriched and impoverished aquaria were associated with differential influences on telencephalic and tectal cell proliferation, **Figure 6A** (Telencephalon) and B (Tectum). Indeed, the average total number of telencephalic cells was greater in EE (1,423,556 ± 93,308) than in IE (mean ± SD: 1,150,320 ± 62,520; two-tailed t-test, p < 0.0007; t = 5.36; n = 5) animals. **Supplementary Tables S1** and **S2** show all stereological parameters and cell count results. Detailed volume estimates of the telencephalon and tecum opticum are shown in **Tables S3** and **S4**, respectively. Comparative analysis of cell counts, and volumes and correspondent statistical results are exhibited in **Table S5**.

### Differential Influences of Contrasting Environments on Peripheral Blood Cell Counts: A Quantitative Flow Cytometry Analysis

**Figure 7:**
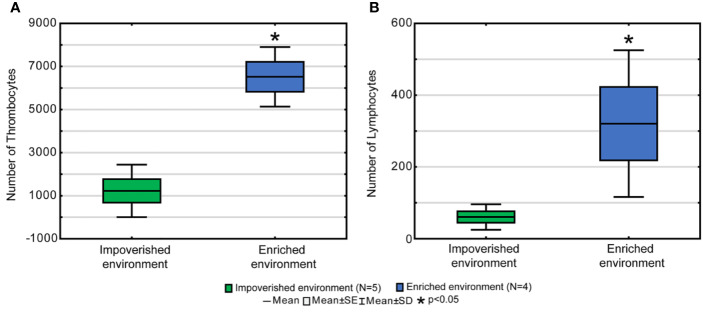
Flow cytometry analysis results of peripheral blood samples of EE and IE fishes. Note that as compared with IE, a significant greater number of thrombocytes **(A)** and lymphocytes **(B)** are observed in EE fish. *p < 0.05.

Another group of adult individuals growing in aquaculture conditions was subjected to physical exercise using running water flow or maintained sedentary. Compared with the sedentary group, the exercised group had a significantly higher density of lymphocytes, and platelets but no significant differences in erythrocytes and granulocytes based on flow cytometry results for peripheral blood (**Figure 7** and **Tables S6** and **S7**).

### Transcriptome Analysis

After obtaining and quantifying the expressed transcripts, we characterized the differential transcript expression associated with each experimental condition. Please follow the link below for all information regarding the transcriptomic analysis: https://dataview.ncbi.nlm.nih.gov/object/PRJNA600147?reviewer=k82rehvn82vea99grmf82igskd.

**Figure 8:**
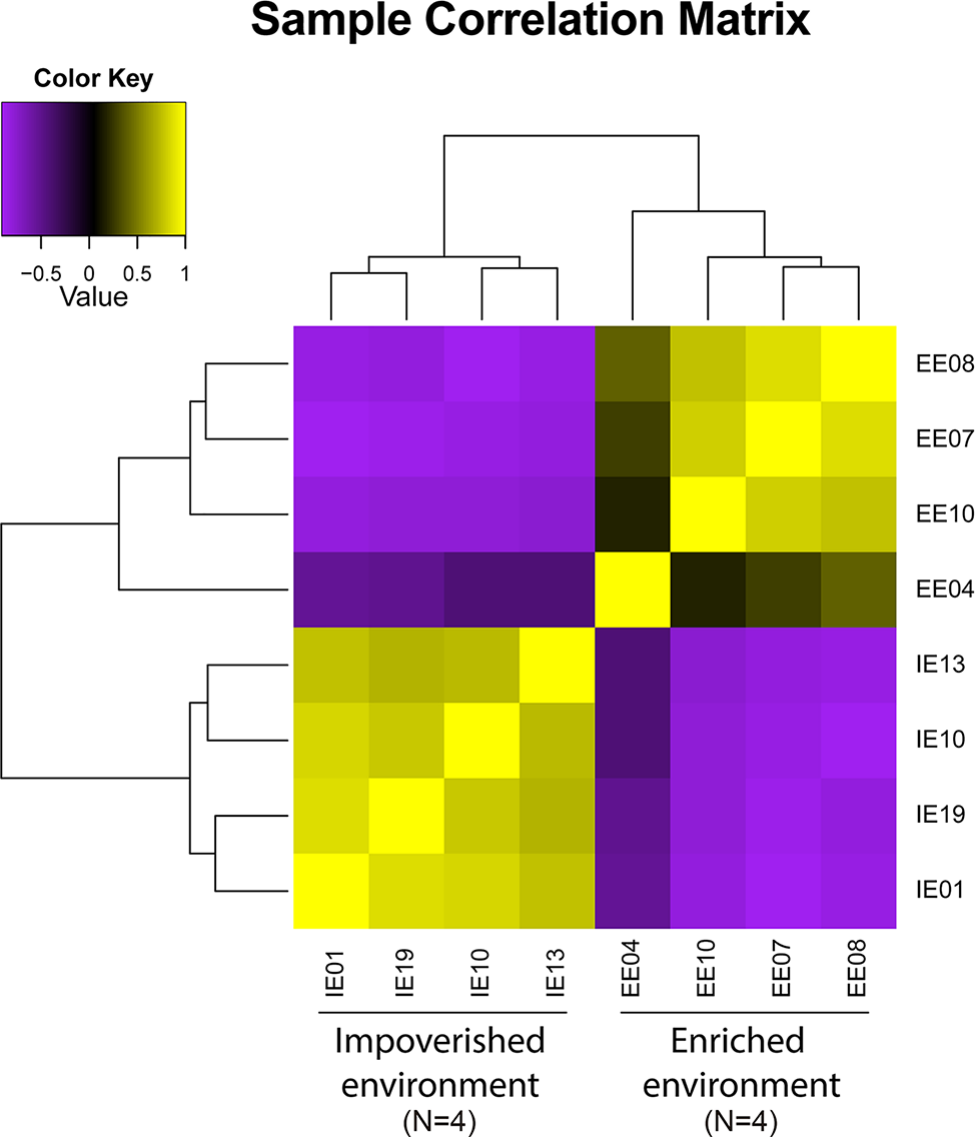
Sample correlation matrix from eight individuals subjected to neurotranscriptome analysis [four from the impoverished environment (IE) and four from the enriched environment (EE)], showing significant levels of coherent clustering between the brain samples of *Colossoma macropomum* raised in contrasting environments.

**Figure 9:**
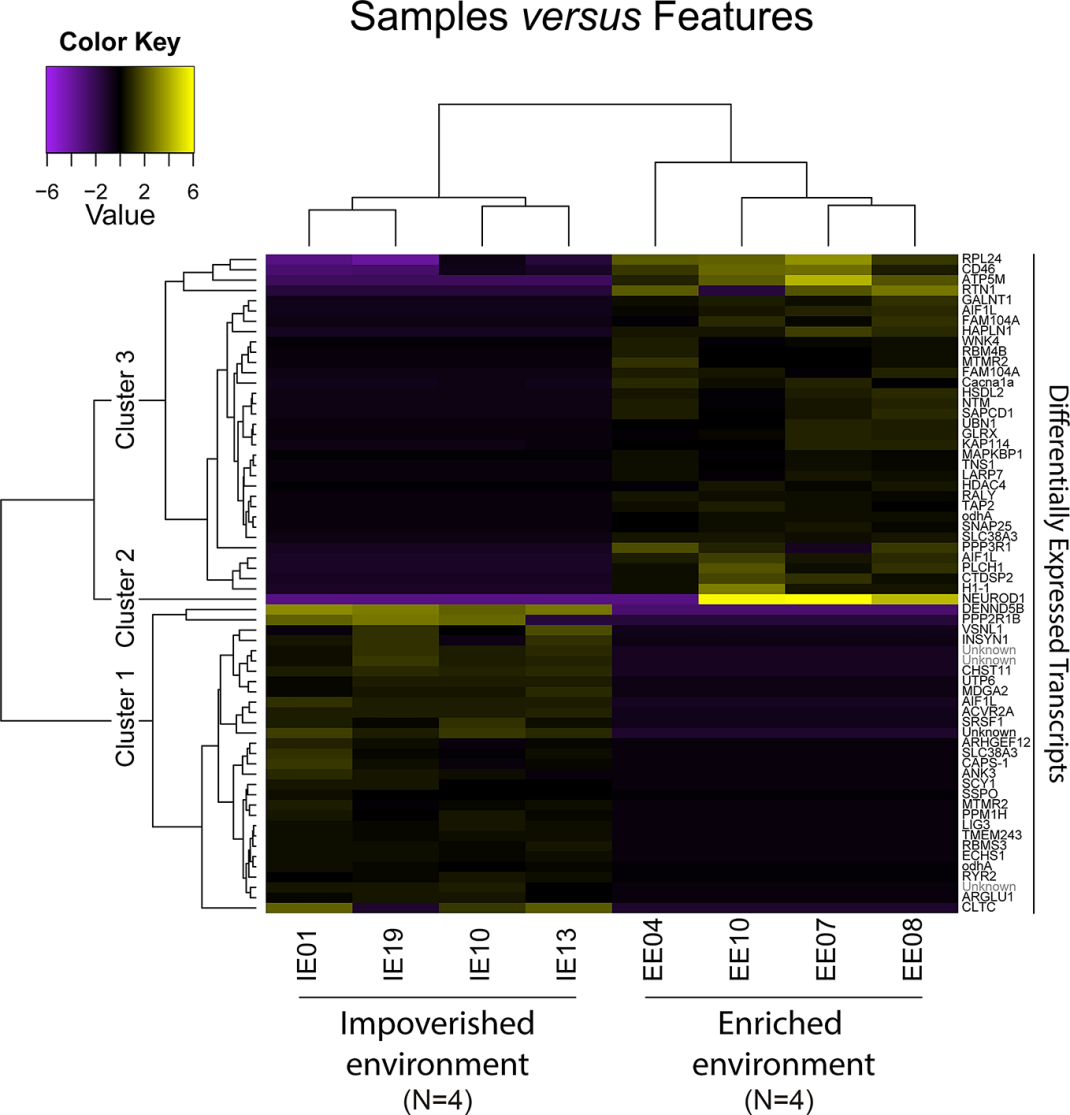
Heatmap from 64 differentially expressed transcripts from *C. macropomum* individuals. Yellow indicates upregulated and purple downregulated transcripts. The upper cladogram represents a cluster analysis between individuals based on transcript expression, and the left cladogram shows the cluster analysis between each transcript. Three main clusters of transcripts with distinct expression profiles are indicated. On the right is the transcript annotation according to best BLAST hit (see **Table S8.xlsx**). “Differentially expressed transcripts”.

**Figure 10:**
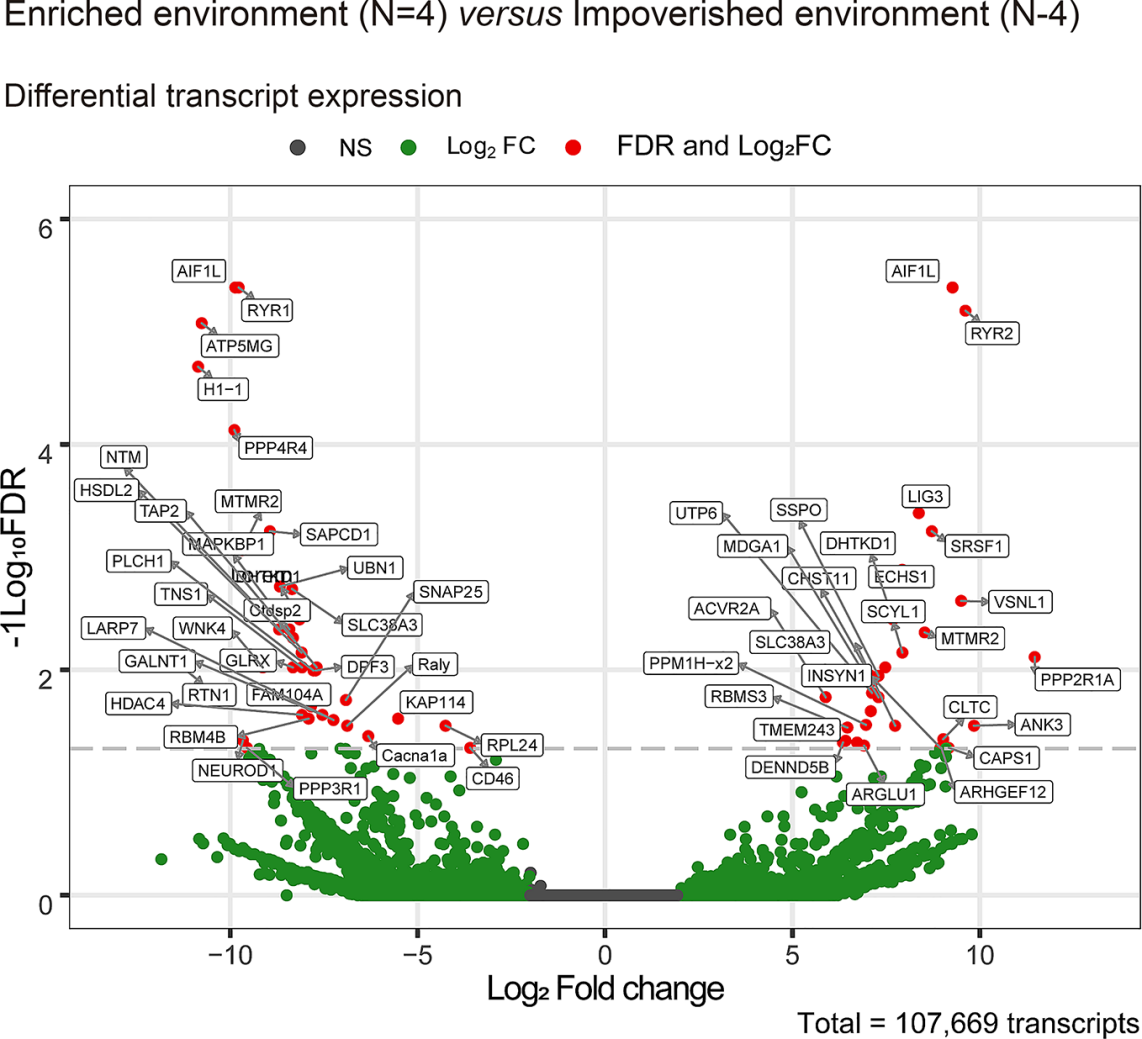
Volcano plot of differentially expressed transcripts in IE and EE groups of *Colossoma macropomum* individuals. Middle compacted grey dots represent non-significant differential expression, green dots indicate transcripts that were differentially expressed for fold change, and red circles, above grey dotted line, correspond to differentially expressed transcripts (−1 < logFC > 1 and −1Log_10_FDR < 0.05). FDR indicates the false discovery rate (rate of false events; p values corrected for multiple comparisons).

The sample correlation matrix from the eight individuals demonstrated significant clustering between the samples (**Figure 8**). We reconstructed a rather large number of transcripts (107,669) that ultimately yielded 64 differentially expressed transcripts between IE and EE fish brains (**Figures 9** and **10**).

We evaluated and compared the general composition of genes in each of the assemblies by performing GO analysis for the main three GO categories (Lees et al., 2012). The histogram in **Supplementary Figure S2** shows the percentage and absolute number of transcripts sharing the same GO term through each group (IE and EE). **Supplementary Table S8** shows differential expression of transcripts for EE and IE individuals, identifying sequence name, gene description, GO identities, GO names, integrated resource of protein families (InterPro) IDs, and InterPro Go names.

### Discussion

Several lines of evidence suggest that teleosts can navigate based on allocentric maps, dependent on the integrity of telencephalon (Salas et al., 1996a; Salas et al., 1996b; López et al., 2000; Rodriguez et al., 2002; Durán et al., 2008; Broglio et al., 2010). Here, we found that compared with individuals maintained for 192 days in an IE aquarium, the total number of cells in the telencephalon of individuals maintained in an EE aquarium was increased. This increase accompanied a better performance on learning and memory plus maze task and differential expression of 64 genes between IE and EE individuals.

### EE and Spatial Learning and Memory

Different from endothermal mammals and birds, ectotherms show cell proliferation resulting from nonspecific influences in the environment that may act indirectly through changes in body temperature (Rieder and Cole, 2002; Radmilovich et al., 2003), sex (Perry and Grober, 2003), age (Zikopoulos et al., 2000; Traniello et al., 2014), and somatic and neural injuries followed by regeneration (Ilieş et al., 2014). These effects may influence cell counts (Dunlap, 2016). To minimize these influences, here, we maintained EE and IE aquaria under controlled experimental conditions, including water temperature, pH, O_2_ concentration, day-light cycle, noise level, age, and number of individuals per volume of water. With this experimental setup, we expected that any significant differences between fish in the EE and IE aquariums in performance on the plus maze task and in numbers of telencephalic cells would be specific. These findings are in line with previous evidence in mammals and birds demonstrating a significant increase in the total number of neurons and glial cells in the hippocampus (Sherry and Macdougall-Shackleton, 2015; Cooper et al., 2018).

As previously indicated, the number of brain cells in teleost fish increases throughout life with age, body weight, and body length, but information about the influences of EE on teleost cell proliferation is scarce. Telencephalic cell proliferation in the forebrain of zebrafish (*Danio rerio*) maintained in an enriched aquarium was found to be greater among cells immunolabeled for proliferating cell nuclear antigen, suggesting that EE may alter cell cycle in zebrafish (Von Krogh et al., 2010b). Similarly, results using bromodeoxyuridine as a cell marker in *Brachyhypopomus gauderio* demonstrated increased cell proliferation throughout the brains of individuals in a wild environment compared to animals maintained in captivity (Dunlap et al., 2011). Finally, compared with individuals maintained in an environmentally simple structure, the salmonid species *Oncorhynchus kisutch* and *Salmo salar* showed an increase in bromodeoxyuridine (Lema et al., 2005) and NeuroD1 mRNA (Salvanes et al., 2013), respectively, in both dorsomedial and dorsolateral telencephalic regions when subjected to an environmentally complex stimuli structure.

Although we did not investigate the subjacent mechanisms in the present report, we expanded our previous observations to *C. auratus* (Abreu et al., 2019), demonstrating that small-scale physical EE also alters cell cycling in the telencephalon of *C. macropomum* and that these changes coincide with enhanced performance on spatial learning and memory.

### Transcriptomic Analysis and Flow Cytometry of Peripheral Blood Samples

Teleostei have been used as well to model a number of issues related to human disease due to preserved genes that have been lost in mice and rats (Ebrahimie et al., 2016; Hin et al., 2020). As an example, zebrafish preserve AD-relevant transcript isoforms of the PRESENILIN genes such as PS2V which shows increased expression in late onset sporadic AD brains (Hin et al., 2020).

Differential upregulation of transcript expression may be an appropriate starting point in the search for potential physiological changes associated with genetic or environmental changes. A good example in the current work is the upregulation of PPP2R1B, part of the serine/threonine (Ser/Thr) phosphatase pathway. This gene, which was previously described in mammals and in zebra fish (Sueyoshi et al., 2012), is associated with protein phosphatase 2, one of the four major Ser/Thr phosphatases. Here, we found upregulation of PPP2R1B in the brains of fish maintained in the enriched aquarium. Activation of this pathway seems to play a key role in many forms of learning and memory (Shioda and Fukunaga, 2017). Ser/Thr phosphorylation and dephosphorylation regulate presynaptic and postsynaptic events in excitatory and inhibitory neurons, including long-term potentiation and long-term depression, which are essential steps of both short- and long-term memory formation (Mansuy and Shenolikar, 2006). Based on previous findings (Hu et al., 2009), individuals maintained in EE show a selective increase in small heat shock protein and pre- and postsynaptic protein expression in the hippocampus, related to synaptic plasticity and learning and memory. If these physiological implications also are valid in other teleosts, such as *C. macropomum*, it is reasonable to propose that the differential expression of selected transcripts described in the present report may be associated with better performance on spatial memory tests in the EE group.

EE induces significant beneficial effects on cognition and MAPK cascade effector mitogen/stress-activated kinase 1 (MSK1) which is a critical regulator of hippocampal progenitor cell proliferation within the subgranular zone, is upregulated in mice maintained in enriched environment (Karelina et al., 2012). In humans, these effects of proliferation and differentiation seem to be expressed through irisin, a myokine released during exercise, which is associated with a series of physiological roles including osteogenesis and cellular proliferation in the hippocampus (Panati et al., 2016; Panati et al., 2018). Coherently, the differential expression of transcripts related to mitogen-activated protein kinase pathways (PPP3R1 and PPP4R4) which respectively regulates calcineurin and phosphatase activity were associated with EE individuals, whereas PPP2R1A which is implicated in the negative control of cell growth and division was differentially expressed in IE subjects; for details, see https://www.genecards.org/.

Similarly, results of peripheral blood sample quantitative analysis using flow cytometry suggested an increased cellularity with a statistically significant greater number of lymphocytes, and platelets in EE fishes. These findings are in line with previous reports dedicated to assessing exercise/EE influence on immune response and brain in humans (Smith, 1997; Walsh et al., 2011) and experimental models (Pflieger et al., 2018).

### Methodological Limitations

To quantify changes in telencephalic number of cells, we applied the optical fractionator, an accurate method of quantification combining properties of an optical dissector and fractionator that has been used in a variety of studies to determine cell numbers in multiple brain regions. The optical fractionator is unaffected by histological changes or shrinkage, an important issue when performing comparative analysis between experimental groups (West et al., 1991; Glaser and Glaser, 2000; West, 2002).

The main variability in the present analysis was biological, with the ratio CE^2^/CV^2^ < 0.5,where CE is the estimated coefficient of error (coefficient of Scheaffer) and CV is the coefficient of variation, i.e., the ratio between CE^2^ and observed variance of the group, CV^2^, should be less than 0.5 (Slomianka and West, 2005). To minimize possible sources of variation, all data were collected and analyzed with the same unbiased methodology and we all samples were obtained using the same tissue processing protocols. Thus, we expected to reduce non-biological sources of errors to acceptable levels.

Fish from different groups were the same age but had distinct body weights and body lengths before sacrifice. Although we offered the same amount of food for IE and EE individuals, we had not either the control of the amount of food that each individual ingested or the ingested amount of microorganisms that are growing on the surface of plants and decorations in EE aquaria. This ingesta without measurements imposes a limitation in the explanations of the significant differences found between the body weight and size of IE and EE individuals. However, because the mean values and standard errors of oxygen were similar (statistical tests did not show any significant differences), we suggest that these variables do not explain the body weight and size differential results. Although we have not controlled motor activity of each individual, the running water flow during 12 h a day in EE aquaria, obliged EE individuals to exercise more than individuals in IE aquaria, where no water pumps were available to oblige to exercise. Thus, it is reasonable to suggest that this difference in the intensity of exercise and visual spatial stimuli may have significantly contributed to the differential effects associated with contrasting environments.

Thus, differential growth occurred under similar conditions for water temperature, pH, O2 concentration, day-light cycle, noise level, and number of individuals per volume of water in the aquarium, which may have minimized potential confounding factors. Because interactions between individuals were potentially similar in both aquariums, water stream (exercise) and visuospatial stimulation in the enriched aquarium may have been the variables that differed between the groups. These differences were on average linked to a higher number of cells in the telencephalon, differential transcript expression and blood sample cellularity, and better performance on the learning and memory task in *C. macropomum* living in the EE.

Finally, we recognized that the transcriptomic analysis would require further validation using real-time PCR before any conclusions can be drawn. However, the association between descriptive neurotranscriptomics with differential changes in spatial memory and learning, peripheral blood sample cell counts, and telencephalic stereological analysis may be a good starting point to open avenues of integrative research that incorporates molecular, cellular, and systemic/behavioral analysis in Teleostei.

### Significance Statement

In this work, we assessed spatial memory and learning, evaluated differential gene expression using brain transcriptomics, counted cells in the telencephalon, and quantified peripheral blood cell samples of the *Colossoma macropomum* raised in an enriched or in an impoverished aquarium. We identified 64 transcripts with differential expression and associated with important pathways that may be involved in brain and blood responses to EE. Compared with IE group, adult EE individuals showed a significantly higher density of thrombocytes, and lymphocytes, based on flow cytometry analysis of peripheral blood, suggesting differential response under the influence of these contrasting environments. Taken together, our findings provide a starting point for exploring potential genetic regulation mechanisms underlying the better learning and memory performance, increased proliferative activity of the telencephalon, and peripheral blood differences in fish raised in an enriched environment that mimics an active life.

## Data Availability Statement

Figshare link (Trinity Assembly) with 64 annotation genes differentially expressed: https://figshare.com/articles/64_DE_genes_EE_IE_Trinity_Assembly_Pereira_et-al_2020-final-anotation_fasta/12345791. Public link of FASTQ files submitted to NCBI SRA: https://www.ncbi.nlm.nih.gov/bioproject/PRJNA600147.

## Ethics Statement

The animal study was reviewed and approved by Ethics Committee on Experimental Animal Research from the Institute of Biological Sciences, Federal University of Pará, Brazil, CEUA-UFPA 3249260617.

## Author Contributions

All authors made substantial contributions to the conception or design of the work; the acquisition, analysis, or interpretation of data for the work; drafting the work or revising it critically for important intellectual content; giving final approval of the version to be published; and agreeing to be accountable for all aspects of the work in ensuring that questions related to the accuracy or integrity of any part of the work are appropriately investigated and resolved.

## Funding

This research was supported by Coordenação de Aperfeiçoamento de Pessoal de Nível Superior (CAPES), the Brazilian Research Council (CNPq) Edital Universal Grant 440722/2014-4 and Grant 302199/2014-4; Fundação Amazônia Paraense de Amparo à Pesquisa (FAPESPA), Grant Centro de Piscicultura do IFPA Campus Bragança and Núcleos Emergentes, Instituto Federal de Educação ciência e Tecnologia do Pará (IFPA), Editais APIPA 2018 e 2019 . DD and CD were supported by Programa PROCAD AMAZÔNIA/CAPES 88887.310939/2018-00.

## Conflict of Interest

The authors declare that the research was conducted in the absence of any commercial or financial relationships that could be construed as a potential conflict of interest.
