# Supplementary material for: Environmental Enrichment Improved Learning and Memory, Increased Telencephalic Cell Proliferation, and Induced Differential Gene Expression in Colossoma macropomum
Source: Front Pharmacol. 2020 Jun 12;11:840. doi: 10.3389/fphar.2020.00840 (PMC7303308; doi:10.3389/fphar.2020.00840)
Supplement: Supplementary file 4 [file Table_1.docx]

Table S1: Stereological parameters for the Nissl stained cells in the left Tectum opticum of *Colossoma macropomum*. ΣQ- = Total number of objects of interest counted using the optical dissector, SSF = Section Sampling Fraction, ASF = Area Sampling Fraction, TSF = Thickness Sampling Fraction, a(frame) = Area of counting frame, A(x,y step) = grid size.

| **Enriched environment** | **a(frame)**  **(µm)** | | **A(x,y step)**  **(µm)** | | **N° Counting**  **Frames** | | **N° Sections** | | **ASF** | | **SSF** | | **TSF** | | **ΣQ‐** | |  |
| --- | --- | --- | --- | --- | --- | --- | --- | --- | --- | --- | --- | --- | --- | --- | --- | --- | --- |
| EE08 | 350x350 | | 50x50 | | 346 | | 15 | | 0.0204 | | 0.3333 | | 0.6024 | | 2400 | |  |
| EE09 | 350x350 | | 50x50 | | 419 | | 9 | | 0.0204 | | 0.3333 | | 0.5076 | | 4227 | |  |
| EE11 | 400x400 | | 50x50 | | 282 | | 11 | | 0.0156 | | 0.3333 | | 0.5848 | | 2591 | |  |
| EE12 | 350x350 | | 50x50 | | 395 | | 10 | | 0.0204 | | 0.3333 | | 0.5208 | | 3436 | |  |
| EE15 | 350x350 | | 50x50 | | 282 | | 9 | | 0.0204 | | 0.3333 | | 0.4808 | | 1747 | |  |
| **Impoverished environment** | | **a(frame)**  **(µm)** | | **A(x,y step)**  **(µm)** | | **N° Counting**  **Frames** | | **N° Sections** | | **ASF** | | **SSF** | | **TSF** | | **ΣQ‐** | |
| IE01 | | 350x350 | | 50x50 | | 303 | | 10 | | 0.0204 | | 0.3333 | | 0.4405 | | 2160 | |
| IE02 | | 350x350 | | 50x50 | | 571 | | 13 | | 0.0204 | | 0.3333 | | 0.5025 | | 4131 | |
| IE13 | | 400x400 | | 50x50 | | 338 | | 11 | | 0.0156 | | 0.3333 | | 0.4831 | | 2282 | |
| IE15 | | 400x400 | | 50x50 | | 365 | | 13 | | 0.0156 | | 0.3333 | | 0.5181 | | 4338 | |
| IE19 | | 350x350 | | 50x50 | | 618 | | 14 | | 0.0204 | | 0.3333 | | 0.5556 | | 5126 | |
