# Supplementary material for: Environmental Enrichment Improved Learning and Memory, Increased Telencephalic Cell Proliferation, and Induced Differential Gene Expression in Colossoma macropomum
Source: Front Pharmacol. 2020 Jun 12;11:840. doi: 10.3389/fphar.2020.00840 (PMC7303308; doi:10.3389/fphar.2020.00840)
Supplement: Supplementary file 5 [file Table_2.docx]

Table S2. Stereological results of Nissl stained cells on the left telencephalon of *Colossoma macropomum*. SCE = Scheaffer coefficient of error, S.D = Standard deviation. S.E = Standard Error. CV = Coefficient of Variation. CVB = Coefficient of biological variation.

| **Enriched environment** | **N° of cells** | **SCE** | **Thickness**  **(µm)** | **Impoverished environment** | **N° of cells** | **SCE** | **Thickness (µm)** |
| --- | --- | --- | --- | --- | --- | --- | --- |
| EE09 | 1,464,870 | 0.0370 | 21.9 | IE01 | 1,168,118 | 0.0400 | 26.5 |
| EE12 | 1,491,352 | 0.0300 | 23.5 | IE02 | 1,097,903 | 0.0270 | 21.1 |
| EE08 | 1,518,637 | 0.0290 | 19.5 | IE13 | 1,152,474 | 0.0260 | 21.3 |
| EE15 | 1,329,953 | 0.0310 | 22.3 | IE15 | 1,088,966 | 0.0380 | 22.5 |
| EE18 | 1,312,970 | 0.0480 | 27.4 | IE19 | 1,244,139 | 0.0270 | 19.8 |
| **Mean** | **1,423,556** | **0.0350** | **22.9** | **Mean** | **1,150,320** | **0.0316** | **22.24** |
| **S.D.** | 95,307.88 |  |  | **S.D.** | 62,520.39 |  |  |
| **S.E.** | 42,622.98 |  |  | **S.E.** | 31,260.20 |  |  |
| **CV** | 0.0670 |  |  | **CV** | 0.0544 |  |  |
| **CV^2^** | 0.0045 |  |  | **CV^2^** | 0.0030 |  |  |
| **CE^2^** | 0.0012 |  |  | **CE^2^** | 0.0010 |  |  |
| **CE^2^/CV^2^** | 0.2733 |  |  | **CE^2^/CV^2^** | 0.3380 |  |  |
| **CV^2^-CE^2^** | 0.0033 |  |  | **CV^2^-CE^2^** | 0.0020 |  |  |
| **CVB^2^(%CV^2^)** | 72.67 |  |  | **CVB^2^(%CV^2^)** | 66.20 |  |  |
