# Supplementary material for: Environmental Enrichment Improved Learning and Memory, Increased Telencephalic Cell Proliferation, and Induced Differential Gene Expression in Colossoma macropomum
Source: Front Pharmacol. 2020 Jun 12;11:840. doi: 10.3389/fphar.2020.00840 (PMC7303308; doi:10.3389/fphar.2020.00840)
Supplement: Supplementary file 6 [file Table_3.docx]

Table S3. Volume estimates for the left Telencephalon (TEL) of *Colossoma macropomum*. CE=Coefficient of error. Vol.=Volume.

| **Enriched environment** | **Estimated Vol. (mm³) TEL** | **CE**  **Gundersen**  **m=1 TEL** | **Impoverished environment** | **Estimated Vol. (mm³) TEL** | **CE Gundersen**  **m=1 TEL** |
| --- | --- | --- | --- | --- | --- |
| EE08 | 9.7188 | 0.0070 | IE01 | 5.1492 | 0.0150 |
| EE09 | 7.1904 | 0.0170 | IE02 | 9.3744 | 0.0120 |
| EE12 | 8.1900 | 0.0090 | IE13 | 8.8956 | 0.0090 |
| EE15 | 7.2240 | 0.0090 | IE15 | 6.5016 | 0.0170 |
| EE18 | 3.9984 | 0.0130 | IE19 | 9.3576 | 0.0110 |
| **Mean** | **7.2643** | **0.0110** | **Mean** | **7.8557** | **0.0128** |
| **S.D.** | **2.0949** | **0.0040** | **S.D.** | **1.9237** | **0.0032** |
| **S.E.** | **0.9369** | **0.0018** | **S.E.** | **0.8603** | **0.0014** |
