# Supplementary material for: Environmental Enrichment Improved Learning and Memory, Increased Telencephalic Cell Proliferation, and Induced Differential Gene Expression in Colossoma macropomum
Source: Front Pharmacol. 2020 Jun 12;11:840. doi: 10.3389/fphar.2020.00840 (PMC7303308; doi:10.3389/fphar.2020.00840)
Supplement: Supplementary file 7 [file Table_4.docx]

Table S4.Volume estimates for the left tectum opticum (TO) of *Colossoma macropomum*. CE=Coefficient of error. Vol.=Volume.

| **Enriched environment** | **Estimated Vol. (mm³) TO** | **CE**  **Gundersen**  **m=1 TO** | **Impoverished environment** | **Estimated Vol. (mm³) TO** | **CE Gundersen m=1 TO** |
| --- | --- | --- | --- | --- | --- |
| EE08 | 7.72 | 0.0090 | IE01 | 6.81 | 0.0080 |
| EE09 | 9.76 | 0.0180 | IE02 | 13.28 | 0.0050 |
| EE11 | 8.51 | 0.0090 | IE13 | 10.28 | 0.0050 |
| EE12 | 9.06 | 0.0120 | IE15 | 11.31 | 0.0120 |
| EE15 | 6.30 | 0.0100 | IE19 | 14.41 | 0.0040 |
| **Mean** | **8.27** | **0.0116** | **Mean** | **11.22** | **0.0068** |
| **S.D.** | **1.33** | **0.0038** | **S.D.** | **2.95** | **0.0033** |
| **S.E.** | **0.60** | **0.0017** | **S.E.** | **1.32** | **0.0015** |
