# Supplementary material for: Environmental Enrichment Improved Learning and Memory, Increased Telencephalic Cell Proliferation, and Induced Differential Gene Expression in Colossoma macropomum
Source: Front Pharmacol. 2020 Jun 12;11:840. doi: 10.3389/fphar.2020.00840 (PMC7303308; doi:10.3389/fphar.2020.00840)
Supplement: Supplementary file 8 [file Table_5.docx]

Table S5. Statistical analysis results of the comparisons of stereological data between Enriched environment *versus* Impoverished environment groups of *Colossoma macropomum* individuals.

| **Enriched environment *versus* Impoverished environment** | | | | | | | | | |
| --- | --- | --- | --- | --- | --- | --- | --- | --- | --- |
| **Variables** | Shapiro-Wilk test | | Fisher's F-test | | | Two-tailed Student'sT-Test | | |  |
|  | W-value | p-value (Two-tailed) | F (Observed) | F (Critical) | p-value (Two-tailed) | t(Observed) | t(Critical) | p-value (Two-tailed) |  |
| Number of cells (Telencephalon) | 0.908 | 0.269 | 0.430 | 9.605 | 0.434 | 5.360 | 2.306 | 0.001 |  |
| Number of cells (Tectum Opticum) | 0.962 | 0.809 | 0.649 | 9.605 | 0.685 | -1.614 | 2.306 | 0.145 |  |
| Telencephalon Volume | 0.927 | 0.451 | 1.186 | 9.605 | 0.873 | -0.465 | 2.306 | 0.654 |  |
| Tectum Opticum Volume | 0.956 | 0.741 | 0.204 | 9.605 | 0.153 | -2.037 | 2.306 | 0.076 |  |
