# Supplementary material for: Environmental Enrichment Improved Learning and Memory, Increased Telencephalic Cell Proliferation, and Induced Differential Gene Expression in Colossoma macropomum
Source: Front Pharmacol. 2020 Jun 12;11:840. doi: 10.3389/fphar.2020.00840 (PMC7303308; doi:10.3389/fphar.2020.00840)
Supplement: Supplementary file 9 [file Table_6.docx]

Table S6. Statistical analysis results of the comparisons of cytometric data between Enriched environment *versus* Impoverished environment groups of *Colossoma macropomum* individuals.

| **Enriched environment *versus* Impoverished environment** | | | | | | | | | |  | |  | |  | |
| --- | --- | --- | --- | --- | --- | --- | --- | --- | --- | --- | --- | --- | --- | --- | --- |
| **Variables** | Shapiro-Wilk test | | Fisher's F-test | | | Two-tailed Mann-Whitney test | | | Two-tailed Student'sT-Test | | | | | |  |
|  | W-value | p-value (Two-tailed) | F (Observed) | F (Critical) | p-value (Two-tailed) | U | U (standardized) | p-value (Two-tailed) | t (Observed) | | t (Critical) | | p-value (Two-tailed) | |  |
| Nº of Lymphocytes | 0.773 | 0.010 | - | - | - | 20 | 2.337 | 0.019 | - | | - | | - | |  |
| Nº of Thrombocytes | 0.915 | 0.355 | 1.292 | 9.979 | 0.783 | - | - | - | 6.108 | | 2.365 | | 0.000487 | |  |
| Nº of Granulocytes | 0.754 | 0.006 | - | - | - | 14 | 0.000 | 0.365 | - | | - | | - | |  |
| Nº Erythrocytes | 0.876 | 0.144 | 0.063 | 9.979 | 0.046 | - | - | - | -1.057 | | 2.637 | | 0.343 | |  |
