# Supplementary material for: Environmental Enrichment Improved Learning and Memory, Increased Telencephalic Cell Proliferation, and Induced Differential Gene Expression in Colossoma macropomum
Source: Front Pharmacol. 2020 Jun 12;11:840. doi: 10.3389/fphar.2020.00840 (PMC7303308; doi:10.3389/fphar.2020.00840)
Supplement: Supplementary file 10 [file Table_7.docx]

Table S7. Descriptive statistics of cytometric data of *Colossoma macropomum* blood analysis. All values are log normalized

|  | **Enriched environment** | | | | | |
| --- | --- | --- | --- | --- | --- | --- |
| **Cells** | Mean | Standard Deviation | Standard Error | 1st Quartile | Median | 3rd Quartile |
| Number of Thrombocytes | 6520.00 | 1385.40 | 692.70 | 5831 | 6566.50 | 7255.50 |
| Number of Lymphocytes | 320.50 | 204.48 | 102.24 | 168.25 | 308.00 | 460.25 |
| Number of Granulocytes | 42.75 | 23.80 | 11.90 | 27 | 40.50 | 56.25 |
| Number of Erythrocytes | 45830.00 | 8211.68 | 4105.84 | 40999.5 | 42632.50 | 47463 |
|  | **Impoverished environment** | | | | | |
| **Cells** | Mean | Standard Deviation | Standard Error | 1st Quartile | Median | 3rd Quartile |
| Number of Thrombocytes | 1223.00 | 1218.63 | 544.99 | 336.00 | 1168.00 | 1473.00 |
| Number of Lymphocytes | 60.40 | 35.53 | 15.89 | 32.00 | 42.00 | 99.00 |
| Number of Granulocytes | 43.60 | 67.36 | 30.13 | 5.00 | 19.00 | 26.00 |
| Number of Erythrocytes | 61943.00 | 32828.32 | 14681.27 | 37943.00 | 62230.00 | 72946.00 |
